# Guideline for the Management of *Clostridium Difficile* Infection in Children and Adolescents With Cancer and Pediatric Hematopoietic Stem-Cell Transplantation Recipients

**DOI:** 10.1200/JCO.18.00407

**Published:** 2018-09-14

**Authors:** Caroline Diorio, Paula D. Robinson, Roland A. Ammann, Elio Castagnola, Kelley Erickson, Adam Esbenshade, Brian T. Fisher, Gabrielle M. Haeusler, Susan Kuczynski, Thomas Lehrnbecher, Robert Phillips, Sandra Cabral, L. Lee Dupuis, Lillian Sung

**Affiliations:** Caroline Diorio, Paula D. Robinson, and Sandra Cabral, Pediatric Oncology Group of Ontario; Caroline Diorio, L. Lee Dupuis, and Lillian Sung, The Hospital for Sick Children; L. Lee Dupuis, University of Toronto, Toronto; Caroline Diorio, McMaster Children’s Hospital, Hamilton; Susan Kuczynski, Ontario Parents Advocating for Children with Cancer, Barrie, Ontario, Canada; Roland A. Ammann, Bern University Hospital, University of Bern, Bern, Switzerland; Elio Castagnola, Istituto Giannina Gaslini, Genova, Italy; Kelley Erickson and Brian T. Fisher, University of Pennsylvania, Children's Hospital of Philadelphia, Philadelphia, PA; Adam Esbenshade, Vanderbilt-Ingram Cancer Centre, Nashville, TN; Gabrielle M. Haeusler, Peter MacCallum Cancer Centre, Melbourne; Gabrielle M. Haeusler, Royal Children’s Hospital, Parkville; Gabrielle M. Haeusler, Paediatric Integrated Cancer Service, Victoria, Australia; Thomas Lehrnbecher, Hospital for Children and Adolescents, Johann Wolfgang Goethe University, Frankfurt, Germany; Robert Phillips, Leeds Teaching Hospital, National Health Service Trust, Leeds; and Robert Phillips, University of York, York, United Kingdom.

## Abstract

**Purpose:**

The aim of this work was to develop a clinical practice guideline for the prevention and treatment of *Clostridium difficile* infection (CDI) in children and adolescents with cancer and pediatric hematopoietic stem-cell transplantation (HSCT) patients.

**Methods:**

An international multidisciplinary panel of experts in pediatric oncology and infectious diseases with patient advocate representation was convened. We performed systematic reviews of randomized controlled trials for the prevention or treatment of CDI in any population and considered the directness of the evidence to children with cancer and pediatric HSCT patients. We used the Grading of Recommendations Assessment, Development, and Evaluation approach to generate recommendations.

**Results:**

The panel made strong recommendations to administer either oral metronidazole or oral vancomycin for the initial treatment of nonsevere CDI and oral vancomycin for the initial treatment of severe CDI. Fidaxomicin may be considered in the setting of recurrent CDI. The panel suggested that probiotics not be routinely used for the prevention of CDI, and that monoclonal antibodies and probiotics not be routinely used for the treatment of CDI. A strong recommendation to not use fecal microbiota transplantation was made in this population. We identified key knowledge gaps and suggested directions for future research.

**Conclusion:**

We present a guideline for the prevention and treatment of CDI in children and adolescents with cancer and pediatric HSCT patients. Future research should include randomized controlled trials that involve children with cancer and pediatric HSCT patients to improve the management of CDI in this population.

## INTRODUCTION

*Clostridium* *difficile* can be a common commensal of the normal GI flora; however, isolates that produce toxin can result in symptomatic infection.^1^ Well-described risk factors^2,3^ for *C. difficile* infection (CDI) include recent antibiotic and chemotherapy exposure^4-6^ and prolonged hospitalization.^5^ As these factors are common in children and adolescents with cancer and pediatric hematopoietic stem-cell transplantation (HSCT) patients, it is not surprising that CDI has emerged as an important health care–associated infection in this population.^7^

Rates of CDI are increasing over time in adults and children,^8,9^ and CDI is now the most common cause of health care–associated infectious diarrhea.^10^ The importance of CDI has also been highlighted with the emergence of North American pulsed-field gel electrophoresis type 1, a more virulent strain associated with higher morbidity and mortality.^11^ In pediatric patients with cancer, CDI has been associated with an increased risk of death.^3^

Given the prevalence, morbidity, and mortality associated with CDI, strategies for prevention and treatment are important. There are several guidelines for CDI management that have been developed but none is focused on pediatric patients with cancer and HSCT patients.^12-14^ Our objective was to create a clinical practice guideline (CPG) for the prevention and treatment of CDI in children and adolescents with cancer and pediatric HSCT patients.

## METHODS

The guideline panel was multidisciplinary and multinational, with representation from pediatric oncology, pediatric infectious diseases, nursing, pharmacy, a patient advocate, and a guideline methodologist (Data Supplement). Panel members were primarily chosen on the basis of relevant publications while considering geographic representation. We followed standard procedures for creating evidence-based CPGs.^15^ No panel member had conflicts of interest that precluded participation in this panel (Data Supplement). The guideline was funded by the Pediatric Oncology Group of Ontario. CPG creation was editorially independent from the funder.

The key clinical question addressed by the CPG was, “What interventions should be administered for the prevention and treatment of CDI in children and adolescents with cancer or pediatric HSCT patients?” Recommendations are intended for children and adolescents up to age 18 years with cancer or undergoing HSCT. Target users of this CPG are pediatric oncology and HSCT physicians; pediatric infectious diseases physicians; other physicians who facilitate care for these patients, such as general pediatric, emergency room, and intensive care unit clinicians and hospitalists; nurse practitioners; nurses; pharmacists; and other health care professionals who manage CDI in this population.

We used the Grading of Recommendations Assessment, Development and Evaluation approach to generate recommendations and assign level of evidence.^16^ Using the Grading of Recommendations Assessment, Development and Evaluation approach, recommendations may be strong or weak. With strong recommendations, benefits clearly outweigh the risks or vice versa. In this case, almost all patients should receive—or not receive—the recommended intervention as a matter of policy. With weak recommendations, the benefits and risks of the intervention are uncertain or are closely matched. In this case, preferences and values will affect intervention administration. In addition to comparative data, we also considered costs, resources, and logistical challenges in formulating recommendations.

The panel was aware that there are few randomized trials conducted in any pediatric population and, in particular, children with cancer and pediatric HSCT patients. Therefore, the published evidence considered for this CPG included randomized controlled trials in both adults and children, regardless of underlying condition. Only randomized data were included because observational data may be more susceptible to bias. When weighing the evidence, the panel considered the directness of the data to children in general and to children with severe neutropenia and immune suppression as a result of cancer treatment in particular. If recommendations relied on adult trials or immunocompetent patients, evidence quality was downgraded because of indirectness.

With the assistance of a library scientist, we searched for randomized trials indexed from 1980 to March 15, 2018, in the following databases: Medline, Medline in-process, Embase, and the Cochrane Central Register of Controlled Trials. Full search strategies are available in the Data Supplement.

Eligibility criteria were defined a priori. We included studies if patients were human participants, it was a fully published randomized trial with a parallel group design, and it evaluated an intervention for the prevention or treatment of CDI. Exclusion criteria were, for prevention interventions, CDI was not a study end point or was reported as an adverse event; and for treatment interventions, the study population was comprised of less than 90% of patients who were determined to have *C. difficile* as a cause of diarrhea. Studies published in any language were evaluated.

Screening of titles and abstracts, review of full articles for eligibility, and data abstraction were performed independently by two investigators (C.D. and P.D.R.). Any disagreements were resolved by a third reviewer (L.S.). Agreement on study inclusion between reviewers was evaluated using the κ statistic. Strength of agreement was defined as slight (0.00 to 0.20), fair (0.21 to 0.40), moderate (0.41 to 0.60), substantial (0.61 to 0.80), or almost perfect (0.81 to 1.00).^17^

Interventions were divided into those for the prevention and those for the treatment of CDI. For prevention studies, the primary outcome was CDI as defined by the presence of diarrhea and a measure of *C. difficile* toxin from stool. Adverse events were also considered. For treatment studies, outcomes were cure at the end of the treatment period, cure at the end of the follow-up period, recurrence, and adverse events. In studies with more than two arms, the most commonly studied intervention was compared with placebo, no therapy, or standard of care. If none of these was present, then the least-intensive treatment was selected as the control arm. In the event that more than one intervention was evaluated at a similar frequency in these multiarmed trials, the one considered by the panel to be the most active was selected as the intervention arm before seeing the data.

Given the potential for adverse infectious events associated with probiotic or prebiotic administration, we also conducted a separate systematic review to describe invasive infection associated with administration in children with cancer and pediatric HSCT patients. The complete search strategy is provided in the Data Supplement. The same general procedures were followed as described previously. Eligibility criteria were as follows: participants were age < 25 years with cancer or undergoing HSCT (threshold related to age categorization in the search databases), there was exposure to a probiotic or prebiotic agent, and adverse events were reported. Outcomes for this analysis were reported infections and whether the study authors’ attribution of the infection to the probiotic or prebiotic was deemed likely or unlikely.

For randomized trial systematic reviews, synthesis was performed when there were at least three studies within a subgroup. Effects were presented as the risk ratio (RR) and the corresponding 95% CIs. Effect sizes were weighted by the Mantel-Haenszel method, and a random effects model was used for all analyses as we anticipated heterogeneity in effects. Meta-analyses were conducted using Review Manager 5.3 (Cochrane Collaboration, Nordic Cochrane Centre). All tests of significance were two sided, and statistical significance was defined as *P* < .05.

We evaluated the risk of bias using the Cochrane Collaboration’s tool for assessing bias in randomized trials.^18^ Publication bias was explored by visual inspection of funnel plots when at least 10 studies were available for synthesis.^18^

We created evidence tables using synthesized results. Tables were reviewed and recommendations debated in a series of conference calls. Iterations of the final CPG were circulated until all authors agreed with its content. A final revised version was not sent to external experts before submission for publication as the guideline panel contained considerable expertise in pediatric CDI. Instead, we used the peer-review process during manuscript submission as a rigorous and efficient approach to external review. A guideline update is planned in 5 years or sooner in the event of the publication of important new information.

## EVIDENCE BASE, RECOMMENDATIONS, AND EXPLANATIONS

**Fig 1.**
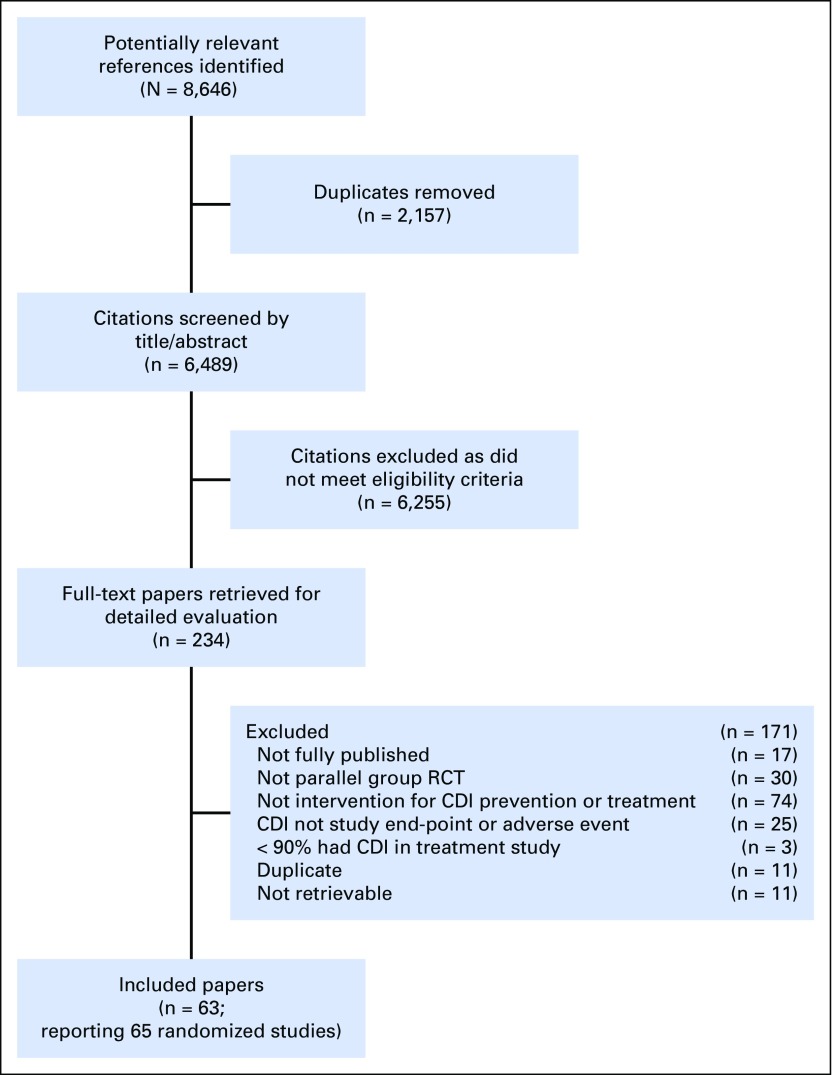
Flow diagram depicting study identification, selection, and reasons for exclusion.

Overall, 63 publications—reporting 65 randomized studies—met the eligibility criteria and provided the main evidence base for this CPG. Figure 1 illustrates the flow diagram of study identification and selection. Agreement in study inclusion between reviewers was almost perfect (κ = 0.96; 95% CI, 0.91 to 1.00). Table 1 lists the characteristics of included trials stratified by prevention or treatment studies and by the specific intervention group. Additional details of the included studies are provided in the Data Supplement. All 18 prevention trials evaluated probiotics or prebiotics. There were 47 treatment trials, of which 45 were in a category amenable to synthesis, namely antibiotics, fecal microbiota transfer (FMT), monoclonal antibodies, and probiotics or prebiotics. Funnel plots did not suggest evidence of publication bias (data not shown). Table 2 lists health questions, recommendations, and remarks in addition to strength of recommendation and level of evidence. Knowledge gaps are listed in Table 3.

**Table 1.**
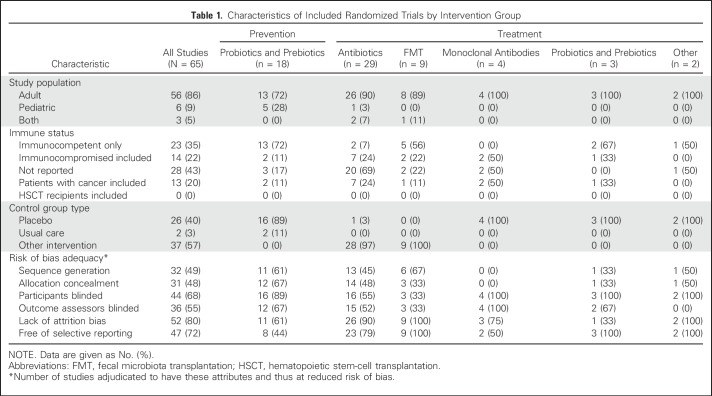
Characteristics of Included Randomized Trials by Intervention Group

**Table 2.**
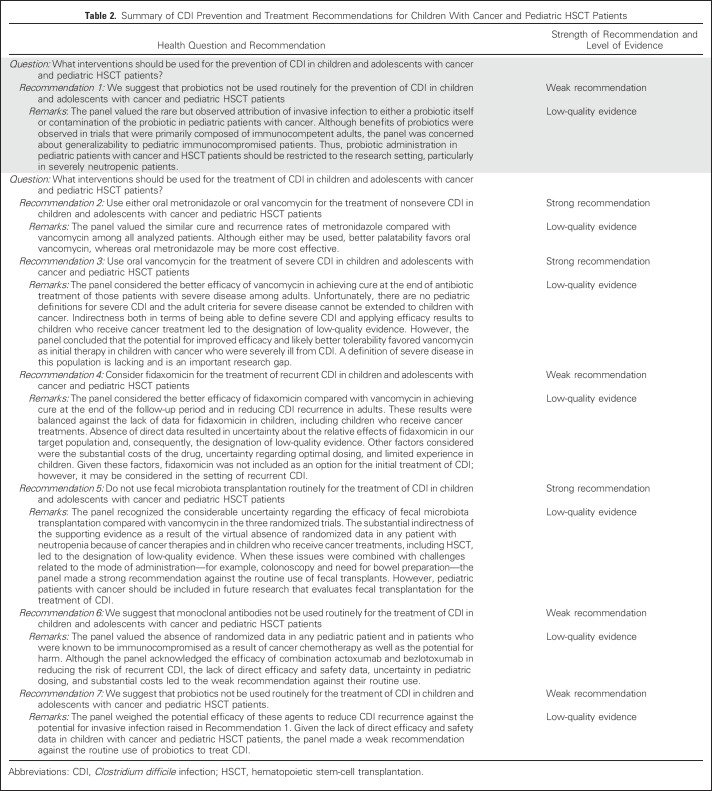
Summary of CDI Prevention and Treatment Recommendations for Children With Cancer and Pediatric HSCT Patients

**Table 3.**
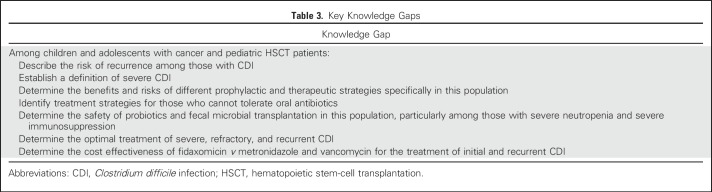
Key Knowledge Gaps

### Recommendation 1

We suggest that probiotics not be used routinely for the prevention of CDI in children and adolescents with cancer and pediatric HSCT patients. (Weak recommendation, low-quality evidence.)

### Literature Review and Analysis

Eighteen studies were included in the analysis of probiotics or prebiotics for the prevention of CDI, with 17 studies of probiotic agents and one study of a prebiotic agent (oligofructose) (Data Supplement). All studies administered probiotics as primary prophylaxis. The most common probiotics studied were *Lactobacillus acidophilus* (n = 7 trials), *Saccharomyces boulardii* (n = 5 trials), and *Lactobacillus rhamnosus* (n = 2 trials). None of the studies included immunocompromised children, and only 22 immunocompromised adults were known to be included across all studies.

When all 18 studies were synthesized, probiotics or prebiotics reduced the risk of CDI (RR, 0.44; 95% CI, 0.28 to 0.70; Table 4). Similar effects were observed when studies were restricted to placebo-controlled trials, probiotic versus placebo trials, and pediatric probiotic versus placebo trials (Table 4). When stratified by probiotic type, significant heterogeneity was not observed (*P*_interaction_ = .37). Table 5 lists the results of the systematic review to assess the safety of probiotic or prebiotic agents in pediatric patients with cancer or HSCT patients. There were three cases of invasive infection attributed by the study authors to a probiotic, namely *Lactobacillus* bacteremia,^21^ absidiomycosis,^24^ and *Saccharomyces* fungemia.^25^

**Table 4.**
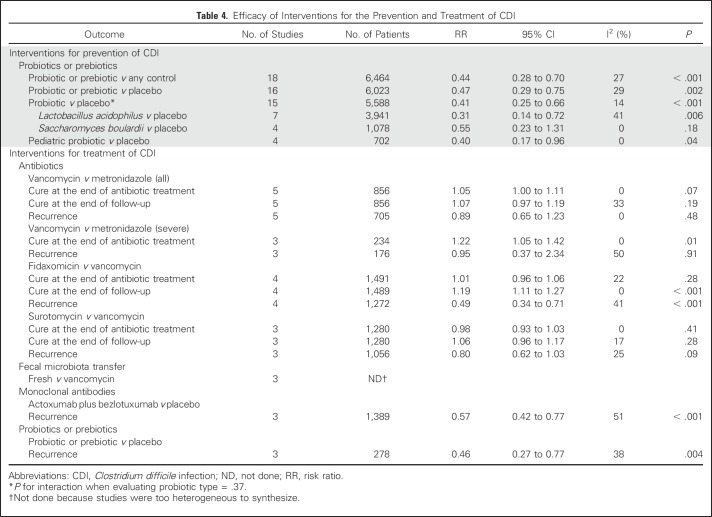
Efficacy of Interventions for the Prevention and Treatment of CDI

**Table 5.**
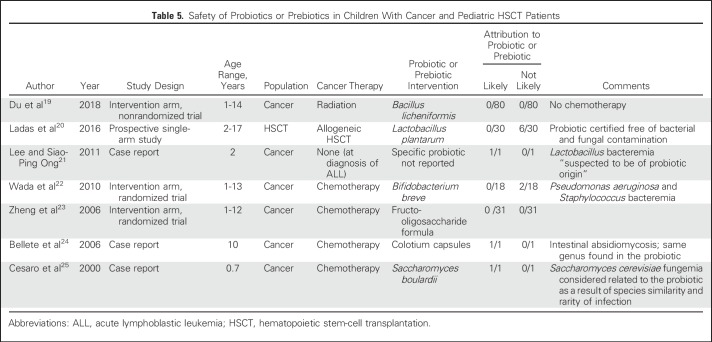
Safety of Probiotics or Prebiotics in Children With Cancer and Pediatric HSCT Patients

The panel valued the rare but observed attribution of invasive infection to either a probiotic itself or contamination of the probiotic in pediatric patients with cancer. Although the benefits of probiotics were observed in trials primarily composed of immunocompetent adults, the panel was concerned about generalizability to pediatric immunocompromised patients. Evidence was considered low quality because the lack of data in neutropenic children with cancer or HSCT recipients resulted in an inability of panel members to conclude that the benefits of probiotics would outweigh safety concerns in our patient population. In particular, the need for an intervention to prevent CDI is greater during profound and prolonged neutropenia when antibiotic exposure and hospitalization are frequent. However, this period may be contemporaneous with a higher risk of developing invasive infection from probiotics. Thus, probiotic administration in pediatric patients with cancer and HSCT patients for primary or secondary prophylaxis of CDI should be restricted to the research setting, particularly in severely neutropenic patients. If a probiotic is administered, a formulation certified as being free of bacterial and fungal contamination is preferred.

### Recommendation 2

Use either oral metronidazole or oral vancomycin for the treatment of nonsevere CDI in children and adolescents with cancer and pediatric HSCT patients. (Strong recommendation, low-quality evidence.)

### Literature Review and Analysis

Among the 29 publications that compared different antibiotics for the treatment of CDI, there were three comparisons with synthesizable data, namely vancomycin versus metronidazole, fidaxomicin versus vancomycin, and surotomycin versus vancomycin (Data Supplement). Fidaxomicin versus vancomycin will be addressed with Recommendation 4.

We identified five trials that compared vancomycin with metronidazole^26-29^ (Table 4 and Data Supplement). None of these studies included children and only two studies included 65 patients with cancer. When these studies were synthesized, vancomycin was not associated with better cure rates at the end of the antibiotic treatment (RR, 1.05; 95% CI, 1.00 to 1.11), cure at the end of the follow-up period (RR, 1.07; 95% CI, 0.97 to 1.19), or recurrence risk (RR, 0.89; 95% CI, 0.65 to 1.23).

Among the three studies that compared surotomycin with vancomycin, there were no significant differences in cure at the end of the antibiotic treatment (RR, 0.98; 95% CI, 0.93 to 1.03), cure at the end of the follow-up period (RR, 1.06; 95% CI, 0.96 to 1.17), or recurrence risk (RR, 0.80; 95% CI, 0.62 to 1.03).^30-32^

The panel valued the similar cure and recurrence rates of metronidazole compared with vancomycin among all analyzed patients in making a strong recommendation that either agent be used for the initial treatment of nonsevere CDI in children with cancer or pediatric HSCT recipients. Better palatability favors oral vancomycin, whereas oral metronidazole may be more cost effective.^33^ An additional consideration is the potential for drug–drug interactions with metronidazole. Oral vancomycin, which is not systemically absorbed, is favored in patients with CDI who receive concomitant medications known or suspected to interact with metronidazole. The panel assigned low-quality evidence because of the absence of direct evidence comparing vancomycin with metronidazole in children with cancer and pediatric HSCT patients. Surotomycin was not included as an option for the initial treatment of CDI because it was not better than vancomycin, there are limited data in pediatric populations, and it is not routinely available for clinical use. Determining the benefits and risks of different antibiotic treatment approaches, particularly among those who cannot tolerate oral antibiotic administration, is an important knowledge gap (Table 3).

This recommendation is focused on children with nonsevere CDI. Unfortunately, definitions for nonsevere and severe CDI have not been established in pediatric oncology. Whereas a definition for severe CDI has been proposed for adult patients, the panel cautions against the generalization of this definition to children with cancer as the adult definition includes parameters, such as higher WBC count and age > 60 years (Data Supplement). On the basis of clinical experience, the panel suggests that severe CDI in pediatric oncology could be defined provisionally as CDI in the presence of toxic megacolon, pseudomembranous colitis, or hemodynamic instability. However, the lack of a robust definition of severe CDI in pediatric cancer and HSCT populations is an important research gap (Table 3).

### Recommendation 3

Use oral vancomycin for the treatment of severe CDI in children and adolescents with cancer and pediatric HSCT patients. (Strong recommendation, low-quality evidence.)

### Literature Review and Analysis

Three of the vancomycin versus metronidazole studies reported outcomes for a subset of patients with severe CDI^26,27^ (Table 4 and Data Supplement). Vancomycin was associated with a significantly higher cure rate at the end of the antibiotic treatment compared with metronidazole among patients with severe CDI (RR, 1.22; 95% CI, 1.05 to 1.42).

The panel considered the better efficacy of vancomycin in achieving cure at the end of the antibiotic treatment of those patients with severe disease among adults. Indirectness both in terms of being able to define severe CDI and applying efficacy results to our population led to the designation of low-quality evidence. Nonetheless, the panel concluded that the potential for improved efficacy and likely better tolerability favored vancomycin as the initial therapy in pediatric patients with cancer who were severely ill from CDI.

### Recommendation 4

Consider fidaxomicin for the treatment of recurrent CDI in children and adolescents with cancer and pediatric HSCT patients. (Weak recommendation, low-quality of evidence.)

### Literature Review and Analysis

We identified five randomized controlled trials that evaluated fidaxomicin. We synthesized four trials that compared fidaxomicin with vancomycin^34-37^ (Table 4 and Data Supplement). None of the studies included children and one study included 75 adult patients with cancer. Whereas fidaxomicin did not result in a higher cure rate at the end of the antibiotic treatment (RR, 1.01; 95% CI, 0.96 to 1.06), fidaxomicin was significantly better than vancomycin in achieving cure at the end of the follow-up period (RR, 1.19; 95% CI, 1.11 to 1.27) and reducing the risk of recurrence (RR, 0.49; 95% CI, 0.34 to 0.71).

The panel considered the better efficacy of fidaxomicin compared with vancomycin in achieving cure at the end of the follow-up period and in reducing CDI recurrence in adults. These results were balanced against the lack of data for fidaxomicin in children, including in children who receive cancer treatments. Absence of direct data resulted in uncertainty about the relative effects of fidaxomicin in our target population and, consequently, the designation of low-quality evidence. Other factors considered were the substantial costs of the drug, uncertainty regarding optimal dosing, and limited experience in children. Given these factors, fidaxomicin was not included as an option for the initial treatment of CDI; however, it may be considered in the setting of recurrent CDI.

Efficacy of fidaxomicin over vancomycin was demonstrated in reducing the risk of recurrence in patients with CDI. Risk of recurrence in pediatric patients with cancer and HSCT patients who experience CDI is uncertain and was identified as an important knowledge gap. Future research should also evaluate the cost effectiveness of fidaxomicin compared with metronidazole and vancomycin for initial and recurrent CDI (Table 3).

### Recommendation 5

Do not use FMT routinely for the treatment of CDI in children and adolescents with cancer and pediatric HSCT patients. (Strong recommendation, low-quality evidence.)

### Literature Review and Analysis

There were nine randomized trials of FMT, of which eight included adults only and one that included both adults and children (Data Supplement). The eight adult trials included a total of seven patients with cancer. The one mixed-age trial included only three children and specifically excluded patients receiving chemotherapy.

These trials used heterogeneous approaches to FMT (fresh and frozen) and different control arms (vancomycin and different types of FMT). Among these studies, the only common approach was fresh FMT versus vancomycin in three studies. These three trials yielded different results,^38-40^ with two being stopped early for efficacy^38,40^ and one for futility^39^ (Data Supplement). There was substantial heterogeneity related to the route of fecal transplant administration, pretransplant therapies (duration of vancomycin and requirement for bowel preparation), and control arms (duration of vancomycin). These issues precluded synthesis (Data Supplement).

The panel recognized the considerable uncertainty with regard to the efficacy of FMT compared with vancomycin in the three randomized trials. The substantial indirectness of the supporting evidence as a result of the virtual absence of randomized data in any patient with neutropenia because of cancer therapies and in children receiving cancer treatments, including HSCT, led to the designation of low-quality evidence. When these issues were combined with challenges related to the mode of administration—for example, colonoscopy and need for bowel preparation—the panel made a strong recommendation against the routine use of FMT. However, pediatric patients with cancer should be included in future research that evaluates FMT for the treatment of CDI.

### Recommendation 6

We suggest that monoclonal antibodies not be used routinely for the treatment of CDI in children and adolescents with cancer and pediatric HSCT patients. (Weak recommendation, low-quality evidence.)

### Literature Review and Analysis

Four randomized controlled trials of monoclonal antibodies for the treatment of CDI were identified.^41-43^ There were no children included in any of these studies, and although two studies included immunocompromised patients, the number who received cancer therapies was not reported.^41^ Three studies that compared a combination of actoxumab and bezlotoxumab with placebo were synthesized. In all three studies, monoclonal antibodies were used as an adjuvant to standard antibiotics, namely vancomycin, metronidazole, or fidaxomicin (Data Supplement). Administration of actoxumab plus bezlotoxumab significantly reduced the risk of CDI recurrence (RR, 0.57; 95% CI, 0.42 to 0.77).

The panel valued the absence of randomized data in any pediatric patient or in patients who were known to be immunocompromised as a result of cancer chemotherapy. They also considered the potential for harm given the systemic administration of these agents. Although the panel acknowledged the efficacy of combination actoxumab and bezlotoxumab in reducing the risk of recurrent CDI, the lack of direct efficacy and safety data, uncertainty in pediatric dosing, and substantial costs led to the weak recommendation against their routine use.

We made an a priori decision as outlined in our methods to evaluate the combination of actoxumab and bezlotoxumab as it was the most commonly studied monoclonal antibody intervention among interventions included in multiarmed trials. However, only bezlotoxumab, and not the combination of actoxumab and bezlotoxumab, is approved by the US Food and Drug Administration^48^ and the European Medicines Agency,^49^ and thus, evaluation of bezlotoxumab alone is of interest. As there were only two studies^41^ available that evaluated bezlotoxumab alone, we could not conduct synthesis.

### Recommendation 7

We suggest that probiotics not be used routinely for the treatment of CDI in children and adolescents with cancer and pediatric HSCT patients. (Weak recommendation, low-quality evidence.)

### Literature Review and Analysis

This recommendation refers to the administration of a probiotic during a CDI episode as an adjunct to CDI-directed antibiotic therapy, not as secondary prophylaxis. Three trials examined probiotics or prebiotics for the treatment of CDI (Data Supplement).^44-46^ No children were included in any of these studies, and only one patient with cancer was included. All studies compared the intervention with placebo when used as an adjuvant to standard antibiotic therapy for CDI. Probiotics or prebiotics significantly reduced CDI recurrence (RR, 0.46; 95% CI, 0.27 to 0.77).

The panel weighed the potential efficacy of these agents to reduce CDI recurrence against the potential for invasive infection raised in Recommendation 1. Given the lack of direct efficacy and safety data in children with cancer and pediatric HSCT patients, the panel made a weak recommendation against the routine use of probiotics to treat CDI.

## DISCUSSION

In this CPG guided by an international multidisciplinary panel, we present recommendations for the prevention and treatment of CDI in children with cancer and pediatric HSCT patients. The panel made strong recommendations to use either oral metronidazole or oral vancomycin for the initial treatment of nonsevere CDI and oral vancomycin for the initial treatment of severe CDI. Fidaxomicin may be considered in the setting of recurrent CDI. The panel suggested that probiotics not be routinely used for the prevention of CDI and that monoclonal antibodies and probiotics not be routinely used for the treatment of CDI. A strong recommendation to not use FMT was made for this population.

A striking finding across the evidence informing this CPG is the lack of high-quality evidence as a result of the omission of pediatric patients with cancer and HSCT patients from randomized trials. Thus, future trials should either focus on this population exclusively or include these pediatric patients in adult trials. Direct data are important. Children who receive cancer therapies will differ from adults with cancer and immunocompetent children in terms of antibiotic exposure, concomitant medications, and comorbidities.^47^ Of importance, children with cancer frequently receive intensive myelosuppressive chemotherapy and most pediatric HSCT procedures are myeloablative. Thus, the safety of any intervention, particularly as it relates to live products, such as probiotics and some FMT procedures, are important to evaluate directly before recommending their routine use.

Some knowledge gaps can be addressed with observational studies and include describing the recurrence rate of CDI and defining and describing those with severe disease in this population. Such studies should be multicenter with large sample sizes to ensure generalizability and precision in estimates. In our review, we found that CDI treatment trials typically reported multiple end points, including cure at the end of the antibiotic treatment period, cure at the end of the follow-up period, and recurrence. The relative importance of these end points is not established and stakeholders may value them differently.

In summary, we present a guideline for the prevention and treatment of CDI in children and adolescents with cancer and pediatric HSCT patients. Future research should define severe CDI and conduct randomized trials that include children with cancer and pediatric HSCT patients to improve the management of CDI in this population.
